# ALD-YOLO: a lightweight attention detection model for apple leaf diseases

**DOI:** 10.3389/fpls.2025.1616224

**Published:** 2025-07-30

**Authors:** Hong Deng, Yiyi Chen, Yilu Xu

**Affiliations:** School of Software, Jiangxi Agricultural University, Nanchang, China

**Keywords:** apple leaf diseases, C2F, attention mechanism, YOLO, object detection

## Abstract

As an important economic crop, apples are significantly affected by disease infestations, which can lead to substantial reductions in apple yield and economic losses. To rapidly and accurately detect apple leaf diseases, we propose a lightweight attention detection model ALD-YOLO based on the YOLOv8 architecture. To improve overall efficiency, we design the Faster_C2F module within the Backbone and Neck by optimizing YOLOv8’s primary C2F (Faster Implementation of CSP Bottleneck with 2 convolutions) modules with the more computationally effective FasterNet Block. To strengthen the model’s ability to capture multi-scale feature information and focus on smaller disease targets, the EMA (Efficient Multi-Scale Attention) module is introduced at the input end where the Neck connects to the detection module of the Head, forming a new Faster_C2F_EMA module. Two novel C2F modules can achieve the optimal balance of detection accuracy and efficiency. Furthermore, to reduce the model’s parameters and retain more image information, most convolution modules in the YOLOv8 architecture are replaced by a lightweight downsampling module ADown. In comparison with YOLOv8n and YOLOv8s, experimental results on the AppleLeaf9 dataset showed that ALD-YOLO increased mAP by 1.4% and 0.6%, and reduced GFLOPs by 29.63% and 79.93%, respectively. The CPU inference testing showed that the improvement of our model in frames per second reached up to 119.23% compared to YOLOv8s. Therefore, our model delivers more stable and efficient detection of apple leaf diseases, even on edge devices.

## 1 Introduction

Globally, apples are one of crucial economic crops with extensive cultivation and enormous market demand. At present, China leads the world in apple cultivation area and production, making apples one of its most important economic crops and fruits. However, as the apple industry rapidly grows and expands, leaf diseases have become a significant factor affecting apple yield and quality. Diseases, such as apple scab, gray spot, apple leaf rust, and powdery mildew, often occur during apple cultivation due to climate or environmental conditions, severely impacting the yield and quality of apples. Consequently, there is a significant demand for accurate methods to identify apple leaf diseases.

Currently, most identification relies on in-field assessments by agricultural experts or experienced farmers’ judgments, which are subjective, time-consuming, labor-intensive, and inefficient. In particular, inexperienced farmers are prone to misdiagnosis and the misuse of pesticides, which not only fail to effectively prevent diseases but also reduce the quality and yield of apples, leading to environmental pollution and unnecessary economic losses. Thus, rapid and accurate identification of apple leaf diseases is of paramount importance.

Since the emergence of the deep convolutional network AlexNet (Krizhevsky et al., 2017), deep learning has made an unprecedented breakthrough in the field of computer vision. Deep learning has been widely applied to the identification of plant diseases owing to its ability to automatically extract image features and its robust performance. Based on AlexNet and GoogLeNet (Liu et al., 2018), proposed a deep neural network model which employed convolutional neural networks to diagnose various leaf diseases. Their experimental results indicated that the proposed CNN-based model attained an accuracy of 97.62% on the test set, which was superior to the traditional methods. Zhong and Zhao (2020) developed a model based on the DenseNet-121 deep convolutional network, which integrated regression, multi-label classification, and focal loss functions to identify apple leaf diseases. This approach identified six types of apple leaf diseases and achieved better results compared to traditional multi-classification methods based on the cross-entropy loss function.

In the realm of accurately localizing apple leaf diseases, mainstream deep learning-based object detection methods are primarily categorized into Two-Stage and One-Stage approaches. Two-Stage methods, such as the R-CNN series (Girshick et al., 2014; Girshick, 2015; Ren et al., 2016), involve two separate network models: one for generating region proposals and another for classifying these regions. This category typically offers high accuracy but low efficiency for large objects and complex scenes. (Bari et al., 2021) developed a Faster R-CNN model for real-time diagnosis of rice leaf diseases, achieving high accuracies: 98.09% for rice blast, 98.85% for brown spot, 99.17% for hispa, and 99.25% for healthy leaves. This model improves traditional methods by providing precise and efficient disease diagnosis. (Wang et al., 2023) proposed a leaf detection method using an improved Faster R-CNN with a CBAM (Convolutional Block Attention Module) (Woo et al., 2018) and DIoU-NMS (Non-maximum suppression). This method achieved an average precision of 95.7%, outperforming Faster R-CNN and YOLOv5 by 2.9% and 7.0%, respectively. Despite the improvement in accuracy, the slow computational speed remains a challenge for real-time detection on the resource-constrained devices, such as the mobile equipment.

One-Stage methods, exemplified by the YOLO (You Only Look Once) series (Redmon, 2016; Redmon and Farhadi, 2017; Farhadi and Redmon, 2018; Bochkovskiy et al., 2020), perform object localization and classification in a single pass with faster speed. However, YOLO algorithms may exhibit higher false detection rates for small objects. To address this problem, Wang et al. (2022) designed the MGA-YOLO network for apple leaf disease, which integrated Ghost modules and the CBAM into YOLO to reduce model size and enhance feature extraction, while also adding an extra prediction layer for large objects. The model outperformed similar methods in accuracy, model size, and speed. However, MGA-YOLO was limited to the whole-leaf recognition, which may result in diagnostic inaccuracies when multiple diseases occur simultaneously on a single leaf. Kumar et al. (2023) utilized an improved YOLOv5 to identify rice leaf diseases by introducing a bidirectional feature pyramid (BI-FAPN) for feature extraction and enhancing detection across different disease scales. Xue et al. (2023) proposed YOLO-Tea which replaced the spatial pyramid pooling fast (SPPF) module in YOLOv5 with the receptive field block (RFB) module, and incorporated self-attention and convolutional block attention modules. This method yielded a 7.6% improvement in the AP@0.5 metric over the original YOLOv5. de Moraes et al. (2023) developed the Yolo-Papaya model by incorporating the Convolutional Block Attention Module into YOLOv7, achieving an mAP of 86.2% across nine categories of papaya fruit diseases. The model maintains a stable number of parameters and inference time compared to other detectors, setting a bench-mark for future research in this area. Ganesan and Chinnappan (2022) proposed a ResNet-YOLO classifier for rice leaf disease detection, which replaced ResNet’s fully connected layer with YOLO. Using preprocessing, adaptive K-means, and FS-SSO techniques, this model showed a 2.08% to 5.3% accuracy improvement over CNN, ResNet, YOLO, and its benchmark model Res-YOLO. However, it required additional image preprocessing and more computational resources and longer training time.

The above literature indicates that deep learning has achieved high accuracy in plant disease detection. However, it is still challenging for deep learning to reduce model parameters, training time, and the need for computational resources and data samples. Ensuring stable inference speed on resource-limited devices and addressing overfitting in case of sample scarcity are also crucial challenges (Turkoglu et al., 2022; Hosny et al., 2023). Li et al. (2024) developed an improved YOLOv8s-based lightweight method for corn leaf disease recognition. However, this method fails to adequately account for the computational interference caused by complex backgrounds in natural scenes, which may comprise recognition performance.

Inspired by these studies, we propose a novel lightweight model based on YOLOv8 for apple leaf disease detection. Our model not only accurately detects small lesions on leaves in natural environments but also ensures practical inference speed on resource-constrained devices by reducing computational complexity.

## 2 Materials and methods

### 2.1 YOLOv8 model

YOLOv8 is a groundbreaking state-of-the-art (SOTA) model that has gained a stellar reputation in the field of object detection. YOLOv8 is available in five versions network depths and parameter counts: YOLOv8n, YOLOv8s, YOLOv8m, YOLOv8l, and YOLOv8x. Considering accuracy, computational speed, and deployment cost, we have chosen YOLOv8s as the baseline model for this study. Based on the advantages of previous YOLO models, YOLOv8 introduces new optimizations aiming at enhancing overall model performance and demonstrating exceptional adaptability to various environmental complexities. YOLOv8 significantly differs from its predecessor, YOLOv5, in the following aspects. Firstly, the C3 (CSP Bottleneck with 3 convolutions) module has been replaced with the C2F module. The C2F module offers a more efficient design by reducing the number of parameters and improving computational efficiency while maintaining strong feature extraction capabilities. Secondly, to avoid the intensive convolution operations during the up-sampling process, the detection head has been restructured using a decoupled architecture, further improving computational efficiency. Additionally, YOLOv8 adopts a flexible anchor-free strategy, which exhibits more stable performance in various object detection tasks compared to the traditional anchor-based approaches. This strategy is particularly important in scenarios that require high detection precision, such as pedestrian and vehicle detection.

**Figure 1:**
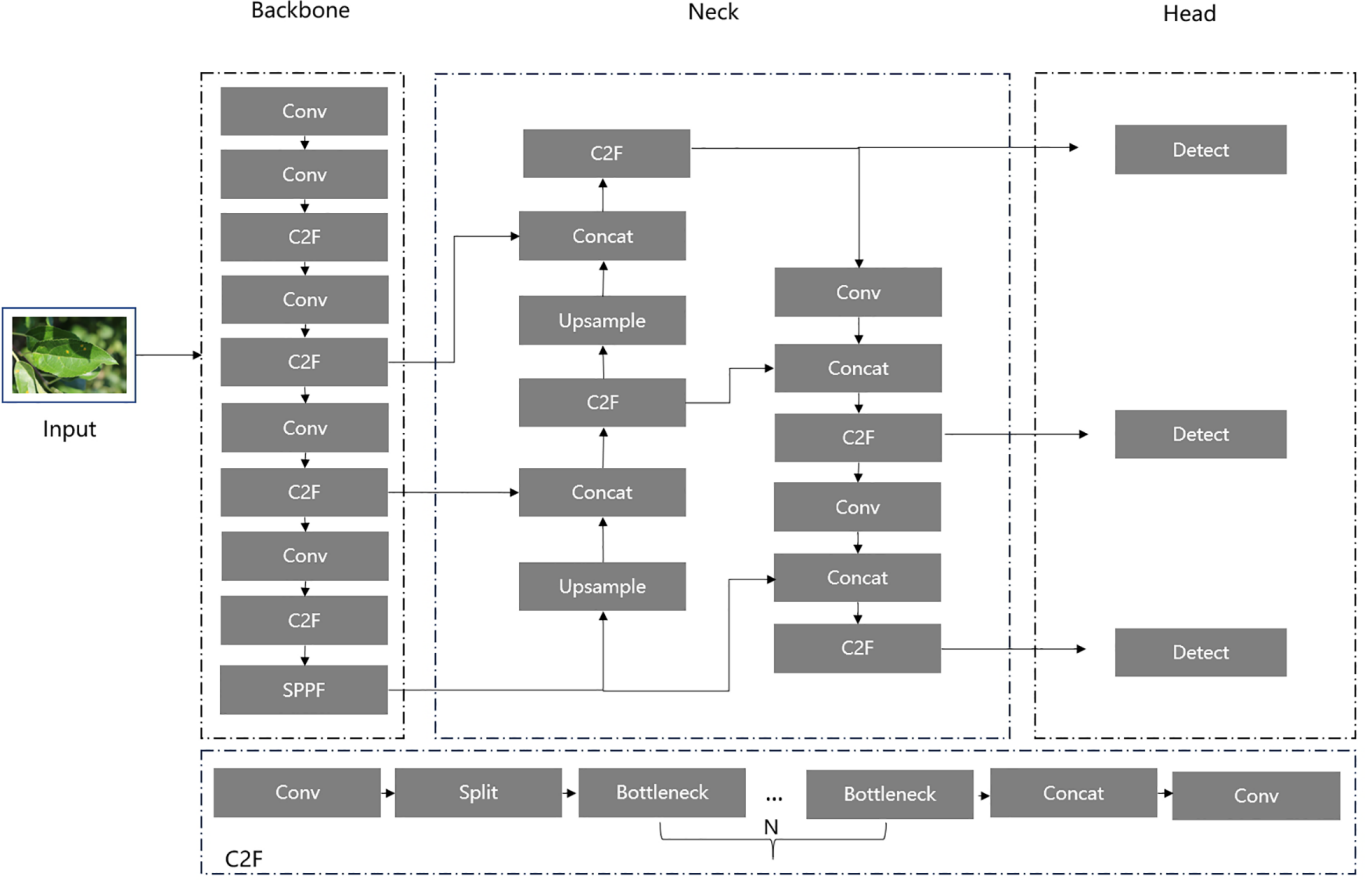
The architecture of YOLOv8 model.

As illustrated in **Figure 1**, the YOLOv8 architecture comprises three components: Backbone, Neck, and Head. The Backbone employs a series of convolutional operations, residual connections, and bottleneck structures to reduce network size and improve performance by extracting raw feature information from input images. The extracted feature information is then passed to the Neck, which further optimizes and refines the features by fusing feature maps from different stages of the Backbone, enhancing feature representation. Finally, the Head processes the feature outputs from the Neck to generate the detailed location and type of detected objects. In our study, we focus on improving the YOLOv8 architecture, particularly its C2F module, to achieve an optimal balance between detection accuracy and computational efficiency.

### 2.2 ALD-YOLO model

**Figure 2:**
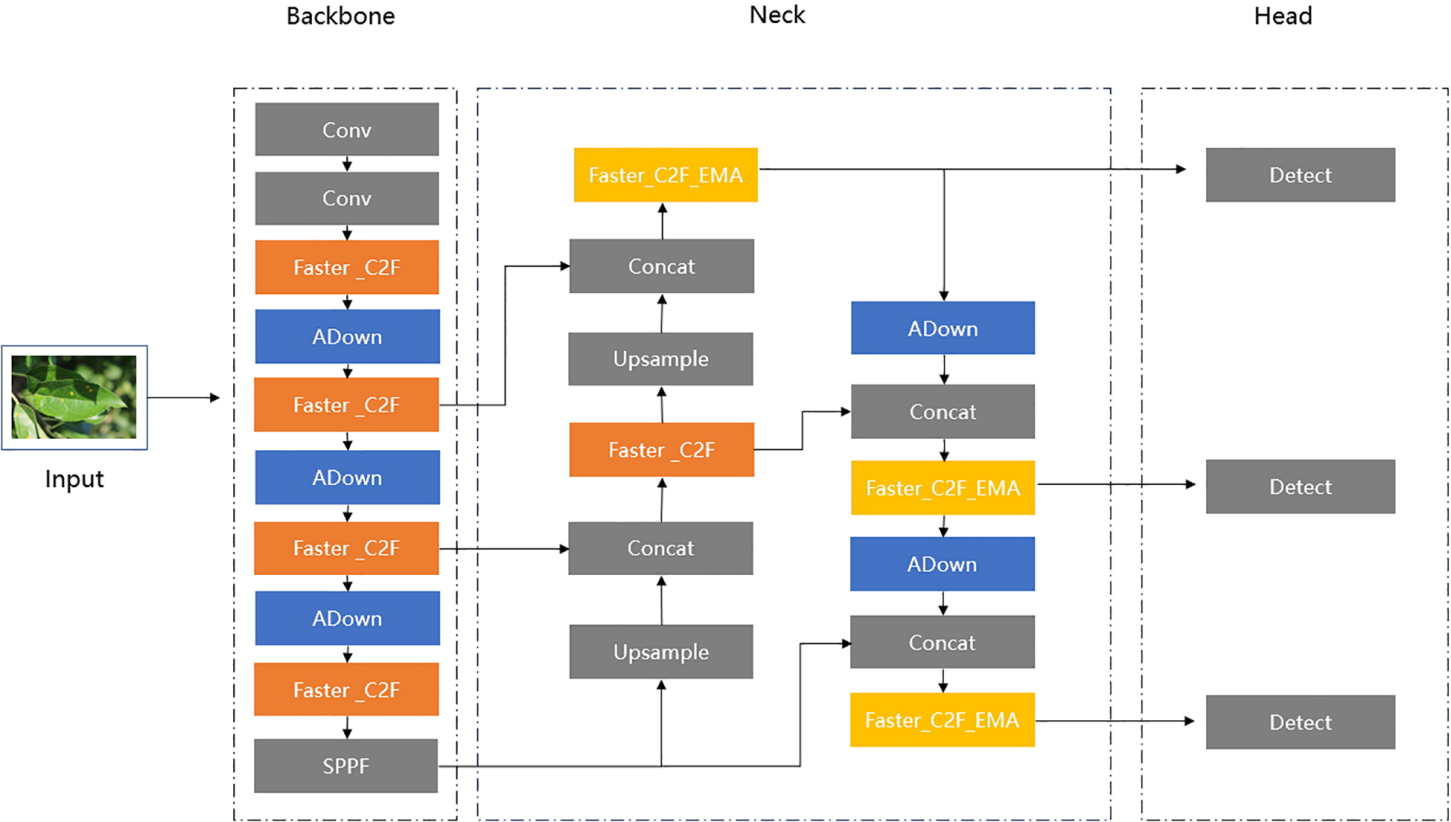
The structure of ALD-YOLO model.

The structure of the proposed ALD-YOLO model is shown in **Figure 2**.

First, we replaced the C2F modules in the Backbone and Neck sections with Faster_C2F, thereby reducing the number of parameters and speeding up computation. This modification is inspired by the FasterNet (Chen et al., 2023) design, which enhances efficient image processing speed by reducing network depth and complexity.

Secondly, we constructed a new Faster_C2F_EMA module in the Neck section by introducing the Efficient Multi-Scale Attention (EMA) (Ouyang et al., 2023) module. By effectively focusing on feature maps with various scales, the EMA module can significantly expand the model’s receptive field. This advantage allows the model to better recognize and process objects with different sizes, which is crucial for object detection tasks. Thus, we incorporated this attention mechanism into the input layers of the Detect components.

Finally, to further optimize the network’s performance, we replaced the convolutional layers in the original YOLOv8 network structure with the ADown module (Wang et al., 2024). The ADown module can not only improve detection accuracy but also significantly reduce computational load. By employing feature map concatenation, the ADown module enhances feature extraction and fusion, providing greater stability when handling complex image tasks. However, considering the large size of the input image, a simple Conv operation provides a more suitable lightweight solution. Therefore, we retained the Conv operations in the first two layers of the Backbone to ensure the initial construction and downscaling of feature maps.

More details can be seen in the following sections.

### 2.3 Faster_C2F module

In the original YOLOv8, the C2F module is a key component that not only facilitates efficient feature extraction but also significantly enhances the model’s detection capabilities through multi-level feature enhancement and fusion strategies. However, despite its excellent performance in feature processing, we identified some limitations, such as the potential redundancy in channel information due to the repetitive stacking of Bottleneck modules. Additionally, the original YOLOv8 tends to miss small objects in complex scenes, such as those with dense objects or partial occlusions.

To balance the speed and accuracy, we replaced the Bottleneck module in C2F module with the core component of FasterNet, known as the FasterNet block. In practical model deployment, we must consider the computational performance of the terminal device. If the model is too complex and computationally demanding, it may not maintain a stable and efficient processing speed. FasterNet is a lightweight neural network model designed specifically for efficient object detection tasks. Partial Convolution (PConv) is a key component of the FasterNet block, which is an efficient method for spatial feature extraction that can significantly reduce redundant computation and memory access.

**Figure 3:**
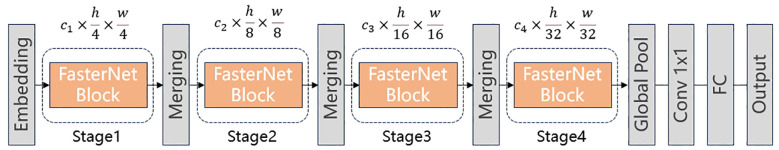
FasterNet structure.

As shown in **Figure 3**, FasterNet consists of an embedding layer, multiple stages with FasterNet blocks, and merging layers, etc. First, the Embedding layer divides the input image into multiple patches. Then, each stage contains a series of FasterNet blocks. And each merging layer is used to reduce spatial dimensions and increase channel depth.

**Figure 4:**
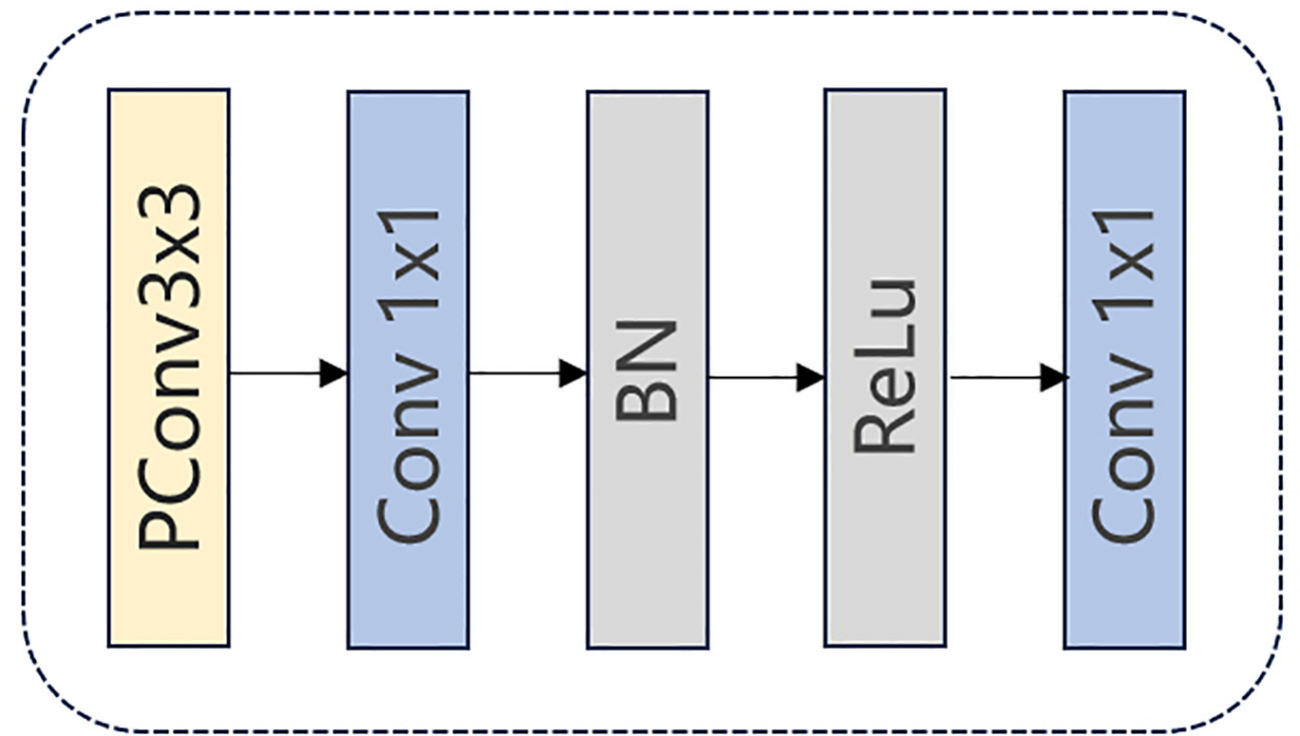
The FasterNet block architecture.

The architecture of FasterNet Block is shown in **Figure 4**. Initially, the input data is fed into a PConv unit where convolutions are applied to only one-fourth of the input feature map’s channels. Subsequently, a 1 × 1 convolution is executed, followed by Batch Normalization and a ReLU activation function. Another 1 × 1 convolution is then performed to make the input and output have the same number of channels. This processing ensures the continuity of information and the integration of multi-scale features, which may improve the model’s performance in complex environments.

With the deepening of convolutional neural network architecture, each successive layer progressively extracts and fuses image features, resulting in deeper feature maps with a substantial amount of highly correlated feature representations. These highly similar features can lead to redundant computations. Additionally, frequent memory access increases latency and consumes more memory bandwidth.

To address these issues, PConv skips convolution operations on certain channels and directly utilizes the redundant information in the feature maps. This strategy can not only reduce the model’s computational demand, which will be suitable for edge devices with constrained computational resources, but also enable the model to capture a broader spectrum of features.

**Figure 5:**
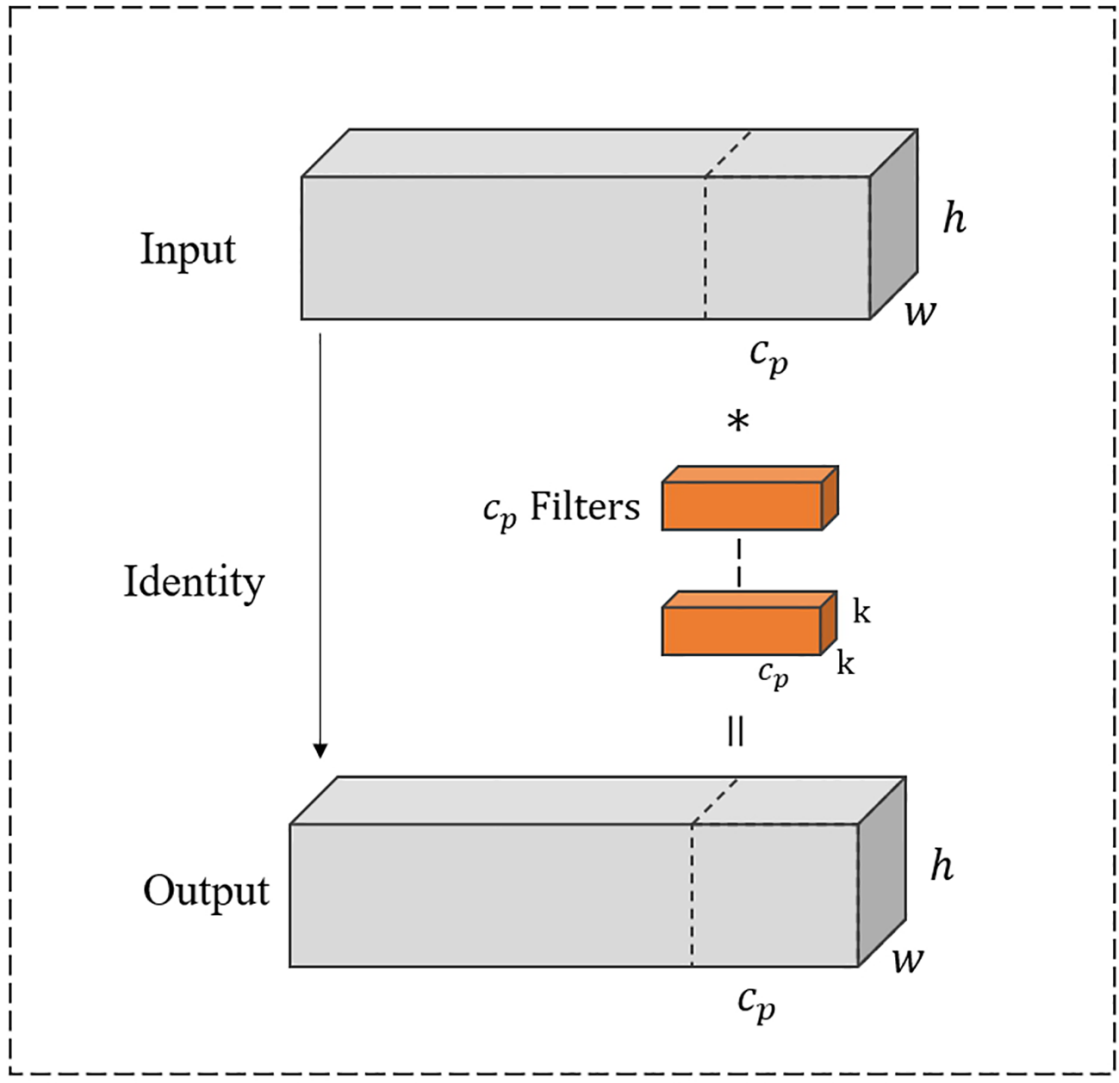
Partial convolution.

In **Figure 5**, PConv divides the input feature map into two segments. One-quarter of the channels are used to refine local features by a 3 × 3 convolution layer, while the remaining three-quarters of the channels preserve the raw feature representations to retain global contextual information without undergoing convolutional processing. Consequently, the processed and unprocessed channels are concatenated altogether.

As shown in **Figure 5**, the input and output channels are the same, where *h* represents the height, *w* represents the width, and $c_{p}$ represents the number of one-quarter of channels. The convolution kernel size is *k* × *k*. Thus, the Floating Point operations (FLOPs) for the PConv operation are calculated as ${h\times w\times k}^{2}{\times c}_{p}^{2}$ .

With a typical partial ratio $r=\frac{1}{\;4}$ , it signifies that the number of channels involved in computation for PConv is only one-quarter of the total number of channels. Therefore, the FLOPs of PConv are notably reduced to 1/16 of those for standard convolution calculations. Likewise, PConv also requires fewer memory accesses as $h\times w\times 2c_{p}+k^{2}\times c_{p}^{2}$ . When the dimension of input significantly exceeds the number of convolution kernel parameters, the memory access of PConv becomes approximately 1/4 of standard convolution.

**Figure 6:**
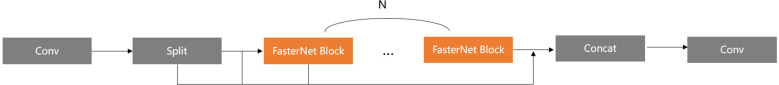
The Faster_C2F structure.

In **Figure 6**, we replace the Bottleneck block with the efficient FasterNet Block to construct the optimal C2F module, namely Faster_C2F. In our new C2F module, after an initial convolution, the input feature map is converted into the intermediate feature map, which is then divided into two parts: one part is passed directly to the subsequent Concat module, while the other part is processed through multiple FasterNet Blocks. The final feature map produced by the FasterNet Blocks is then concatenated with the unprocessed feature map in the Concat module. The concatenated feature map is subsequently passed through a final convolution for further processing, resulting in the final output feature map.

The Faster_C2F structure reduces the overall parameter count and improves computational efficiency compared to the previous design due to the use of FasterNet Block. Consequently, all C2F modules of the network architecture are replaced with the proposed Faster_C2F module, except for the input layers of the Head.

### 2.4 Faster_C2F_EMA module

**Figure 7:**
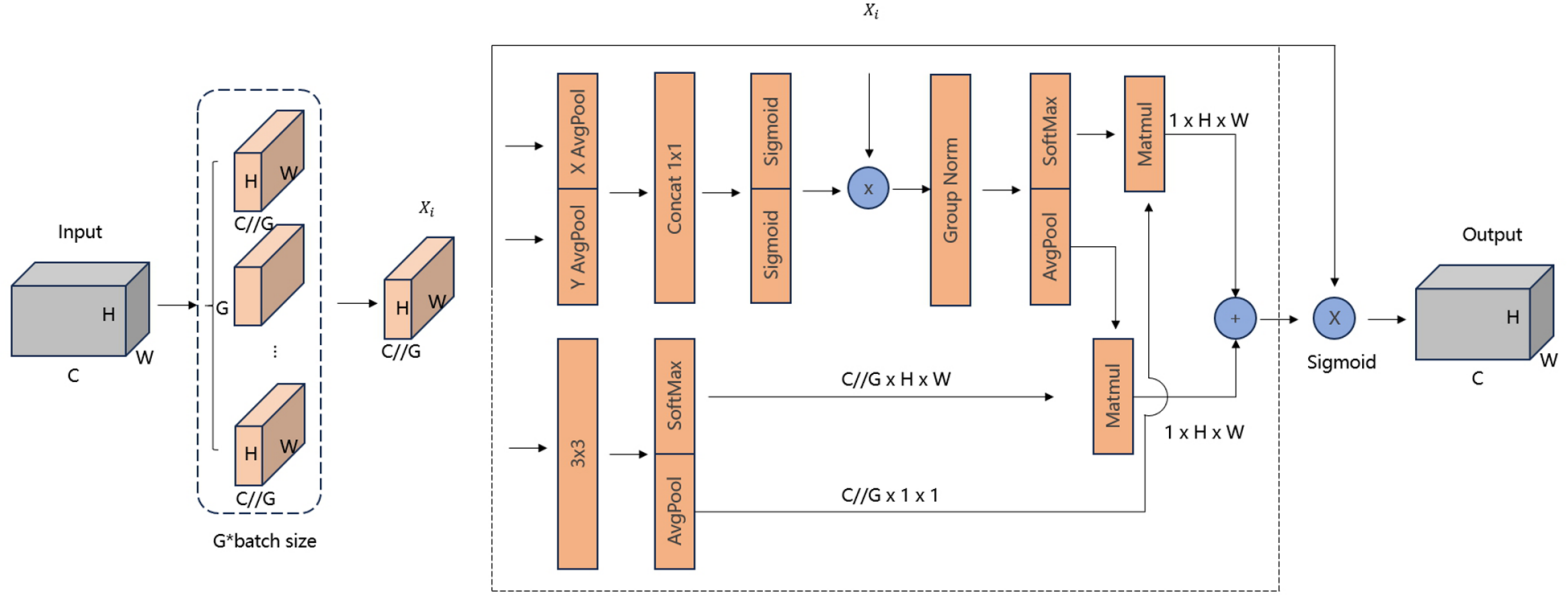
The structure of efficient Multi-Scale attention.

To enhance the representation ability of the model and address the issue of feature loss in small-scale objects, we introduced an attention mechanism into the Neck part of the model, thereby constructing a new C2F module. The EMA module is a novel attention mechanism specifically designed for computer vision tasks such as object detection and image classification. The core idea of this module is to establish both short and long-range dependencies through multi-scale parallel sub-networks. In **Figure 7**, the EMA module divides the input feature map $X\in {\mathbb{R}}^{C\times H\times W}$ into G sub feature maps, where *C* denotes the number of channels, *H* and *W* represent the height and width, respectively, and *G* means the number of sub feature maps. Then, each sub-feature map $X_{i}\in {\mathbb{R}}^{C/G\times H\times W}$ is fed into a triple-branch network in which two 1 × 1 convolutional kernels and a 3×3 convolutional kernel are operated in parallel.

In the 1 × 1 convolutional branch, global average pooling is first applied to encode the channel information of the input feature map across two spatial dimensions (height and width), followed by a shared 1x1 convolution and a Sigmoid function for the first spatial attention map.

In the 3 × 3 convolutional branch, global average pooling is used after the convolution operation to encode the global spatial information of the entire feature map and convert it to match the size of the channel features. The processed feature map is then combined. with the one generated by the 1 × 1 convolutional branch to produce the second spatial attention map.

Finally, the two attention maps from the two branches are summed and then element-wise multiplied with the original feature map, allowing the model to capture pixel-level pairwise relationships and emphasize the global context of all pixels.

Overall, by integrating information across different spatial dimensions, EMA facilitates feature interactions over larger spatial ranges while retaining positional information. It enhances model performance while maintaining low computational overhead, offering higher efficiency compared to other attention mechanisms. This makes EMA particularly suitable for deployment on resource-constrained devices.

**Figure 8:**
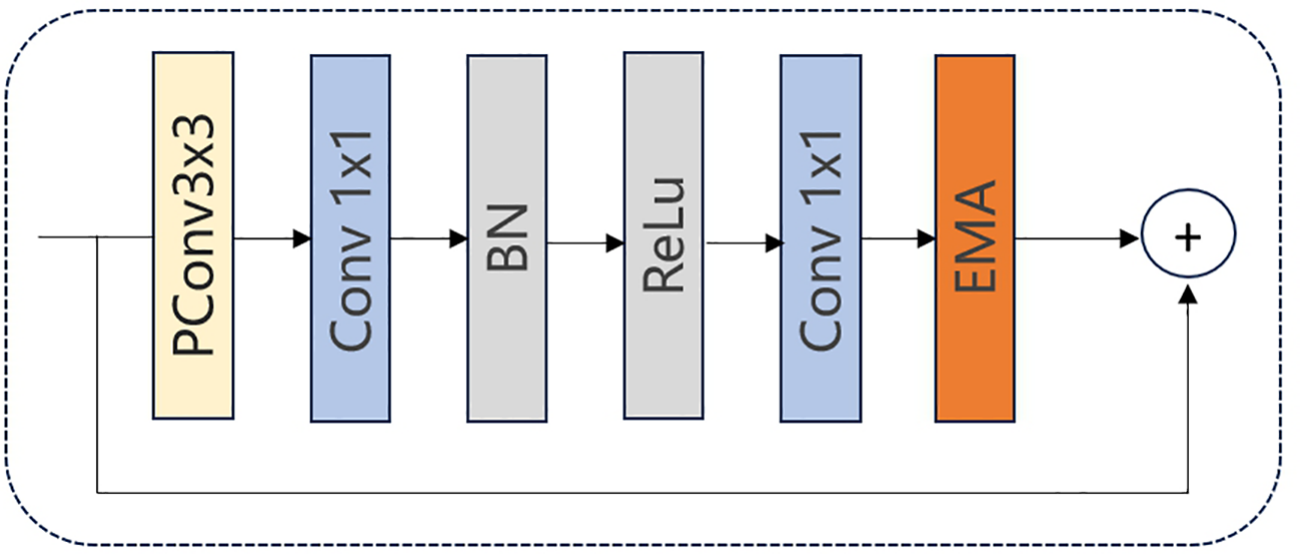
FasterNet-EMA block.

As illustrated in **Figure 8**, we integrate the EMA mechanism into the FasterNet Block to construct a new FasterNet-EMA block. By incorporating the EMA module, we achieve cross-spatial information aggregation and establish long- and short-term dependencies. This enables the acquisition of multi-scale representations and enhances the extraction capabilities for smaller objects.

**Figure 9:**
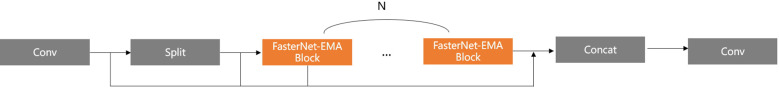
Faster_C2F_EMA.

In **Figure 9**, based on Faster_C2F mentioned above, the new Faster_C2F_EMA module is constructed by substituting the FasterNet Block with the FasterNet-EMA Block. In comparison to the original Faster_C2F, the incorporation of the EMA attention mechanism facilitates the extraction of more comprehensive feature information. However, considering that excessively incorporating EMA attention may not necessarily lead to further improvements in accuracy and could instead affect computational efficiency, we only replaced the original C2F module with the Faster_C2F_EMA block at the input end connecting the Neck and Head.

### 2.5 ADown module

**Figure 10:**
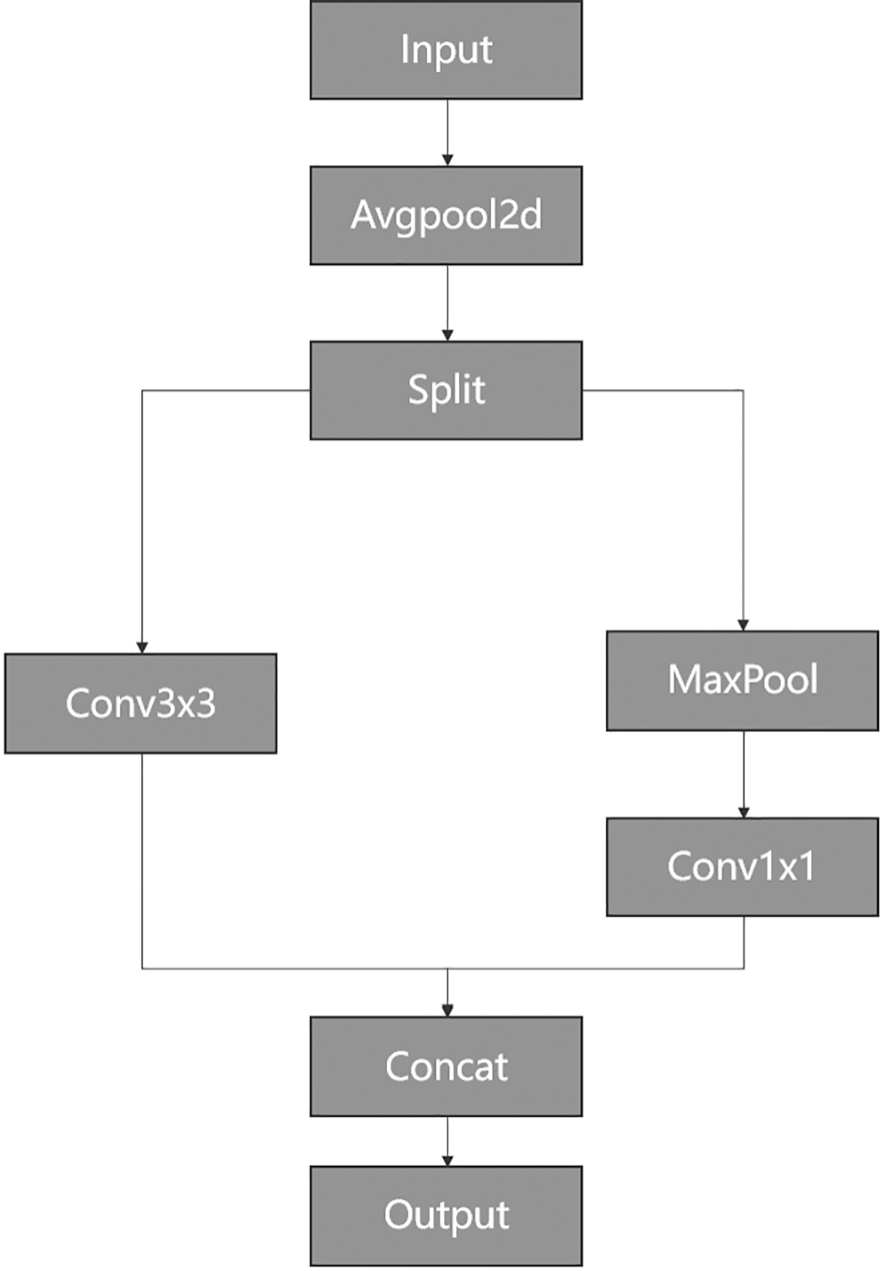
The Adown structure.

Downsampling is a crucial technique in deep learning models that reduces the spatial dimensions of feature maps, enabling networks to extract image features at more abstract levels while alleviating computational burdens. Thus, as shown in **Figures 1** and **2**, the most Conv modules in our network are replaced by a special ADown module used for downsampling operations. In **Figure 10**, ADown employs a dual-path architecture aimed at optimizing the information extraction process through differential processing.

The input feature map is split along the channel dimension into two parts, each of which undergoes distinct convolutional operations.

The first part focuses on local information extraction. To achieve this, it processes the input feature map using a 3 × 3 convolution operation with a stride of two. This design ensures attention to local structures and details. It can effectively retain and capture local features even when reducing spatial resolution. Consequently, this part emphasizes the retention of detailed information during downsampling, while extracting rich local features.

The second part emphasizes the maintenance of global features. Initially, a 3 × 3 max pooling operation is applied, followed by a 1 × 1 convolution operation with a stride of 1. Keeping the stride at 1 preserves the original spatial resolution, mitigating potential loss of important image information during downsampling and ensuring the preservation of global features throughout the process.

Finally, the results of these two convolutional operations are concatenated along the channel dimension. This concatenation combines features from different branches, resulting in a final feature map that retains fine-grained local features during downsampling.

Compared to the original YOLOv8 convolution module, this design strategy not only enhances the model’s representational capacity but also optimizes the utilization of computational resources, leading to a balance between detection accuracy and computational efficiency.

### 2.6 Apple leaf dataset

To validate our method, we selected AppleLeaf9 (Yang et al., 2022) as our benchmark dataset. You can download it from the following link (https://github.com/JasonYangCode/AppleLeaf9). It consists of four different datasets, covering eight types of typical apple leaf diseases. Notably, 94% of the images collected in outdoor environments.

**Figure 11:**
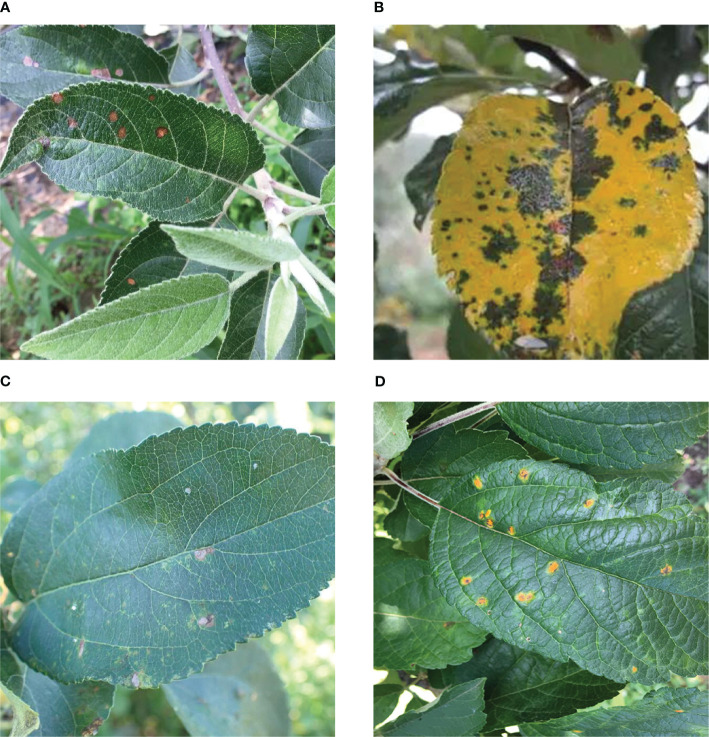
Four types of apple leaf diseases. (**(A)** Alternaria leaf spot. Lesions appear ochre to dark brown, developing ovoid or elliptical shapes with defined margins. **(B)** Brown spot. Initial symptoms manifest as minute purplish-brown to deep brown blisters on leaf surfaces. They become progressively yellow with disease advancement. **(C)** Grey spot. Fungal lesions are characterized by gray or grayish-brown centers with distinct dark brown edges. **(D)** Rust. The characteristic spots have yellow-brown centers with pale yellow chlorotic halos. Corresponding raised pustules form on the surface of the dorsal lobe.).

As shown in **Figure 11**, four typical diseases (Rust, Grey spot, Brown spot, and Alternaria leaf spot) are selected from the dataset, since their pathological features are smaller in size than those of other diseases. Therefore, it is suitable to use them to validate our model. A total of 1146 images were randomly selected from the dataset, and were then allocated to the training, testing, and validation sets in the ratio of 8:1:1. The diseased regions on the leaves were annotated using LabelImg. All visible target object instances within the scene were labeled, with dual verification for data annotation.

## 3 Results

### 3.1 Experimental setup

The experiments were conducted on the following operating platform:

- Hardware: NVIDIA GeForce RTX 4070 12GB GPU, RAM 32 GB;
- Software: Windows 11, Python version 3.8, CUDA 11.8, PyTorch version 2.0.1, and Ultralytics YOLOv8 version 8.0.184.

During training, we utilized the default Mosaic data augmentation, which randomly selects four original images, scales each by a random ratio, and combines them to generate a new composite image. The experimental setup was configured with 100 epochs and a batch size of 32. The initial learning rate (lr0) was set as 0.01, and the weight decay was 0.0005. We employed the Stochastic Gradient Descent (SGD) optimizer and enabled Automatically Mixed Precision (AMP), which contributes to improved training speed and accuracy. Specific parameters are given in **Table 1**.

**Table 1:** Training parameters.

Parameter	Value
Epochs	100
Batch	32
Image_size	640
Optimizer	SGD
Amp	true
lr0	0.01
lrf	0.01
momentum	0.937
nbs	64
weight_decay	0.0005

### 3.2 Experimental analysis

To validate the effectiveness of the proposed model, we trained the dataset on similar YOLO models with consistent hyperparameters. The comparative models included YOLOv6, YOLOv7, YOLOv8, YOLOv9, and the latest YOLOv10. We evaluated the models using the following metrics: Precision (P), Recall (R), mean Average Precision (mAP), Giga Floating Point Operations per second (GFLOPs), and model weights. These evaluation metrics are defined as follows (Equations 1–4):


(1) $$\begin{array}{c} \;Precision\;=\frac{TP}{TP+FP} \end{array}$$

(2) $$\begin{array}{c} \;Recall\;=\frac{TP}{TP+FN} \end{array}$$

(3) $$\begin{array}{c} AP=\int_{0}^{\;1}P\left(R\right)dR \end{array}$$

(4) $$\begin{array}{c} mAP=\frac{\sum_{i=1}^{K}AP_{i}}{K} \end{array}$$

where *TP* (True Positive) means the number of correctly classified positive samples, *FP* (False Positive) means the number of misclassified negative samples, and *FN* (False Negative) refers to the number of incorrectly classified positive samples. *K* denotes the number of classes. $AP_{i}$ represents the AP value for the i-th class.

In **Table 2**, our model shows the superior performance over other comparable models in various metrics except for the slightly lower Precision. Specifically, in terms of the mAP metric, our model shows improvements of 1.4% and 0.6% over the YOLOv8n and YOLOv8s, respectively. For the Recall metric, our model surpasses YOLOv8n and YOLOv8s by 2% and 0.7%, respectively. Especially, compared to the latest YOLOv9 and YOLOv10, our model takes an obvious advantage. In addition, we attempted to replace the original backbone network of YOLOv8 with some lightweight network models, such as MobileNetV3 (Howard et al., 2019) and StarNet (Ma et al., 2024).

**Table 2:** Performance comparison with other models.

Model	Precision	Recall	mAP@0.5	GFLOPs	Weights(M)
YOLOv6n	87.8%	81.9%	88.9%	11.4	10.214
YOLOv6s	87.1%	82.2%	87.8%	45.17	39.697
YOLOv7t	88.1%	88.2%	89.4%	13.2	12.013
YOLOv8n	89.5%	88.3%	91.7%	8.1	6.107
YOLOv8s	90.1%	89.6%	92.5%	28.4	21.481
YOLOv8-MobileNetv3	86.7%	84.5%	87.3%	6.1	6.22
YOLOv8-StarNet	88.9%	86.5%	88.1%	6.5	4.45
YOLOv9t	87.5%	85.4%	88.9%	10.7	5.973
YOLOv10n	84.6%	84.2%	87.5%	6.7	5.631
Ours	89.5%	90.3%	93.1%	5.7	3.953

More critically, our model achieves high accuracy without losing computational efficiency or model size. With a computational complexity of 5.7 GFLOPs and a model size of 3.953 M, our model is more efficient than its counterparts. Such lower computational complexity and smaller model size indicate that our model is more practical for resource-constrained devices.

As shown in **Table 2**, our model demonstrated highest mAP@0.5 score on average. In **Table 3**, we further present the mAP@0.5 values for different disease categories. For Alternaria leaf spot detection, our model achieved better mAP@0.5 value than other models. Notably, during different stages of disease development, some leaf diseases, such as Alternaria Leaf Spot and Grey Spot, present similar symptoms. However, our model can effectively distinguish between these diseases, minimizing diagnostic errors and providing farmers with more reliable decision-making support.

**Table 3:** The mAP@0.5 for different disease categories.

Model	Rust	Grey spot	Brown spot	Alternaria leaf spot
YOLOv6n	95.4%	75.9%	99.2%	85.0%
YOLOv6s	95.5%	72.8%	98.5%	84.3%
YOLOv7t	95.0%	74.7%	99.2%	88.5%
YOLOv8n	95.9%	84.7%	98.4%	88.0%
YOLOv8s	96.4%	87.2%	97.9%	88.6%
YOLOv8-MobileNetv3	93.8%	70.0%	98.0%	87.3%
YOLOv8-StarNet	95.4%	79.1%	99.2%	78.9%
YOLOv9t	94.3%	76.8%	99.3%	85.3%
YOLOv10n	93.4%	75.6%	96.6%	84.4%
Ours	95.3%	88.7%	97.4%	90.9%

**Figure 12:**
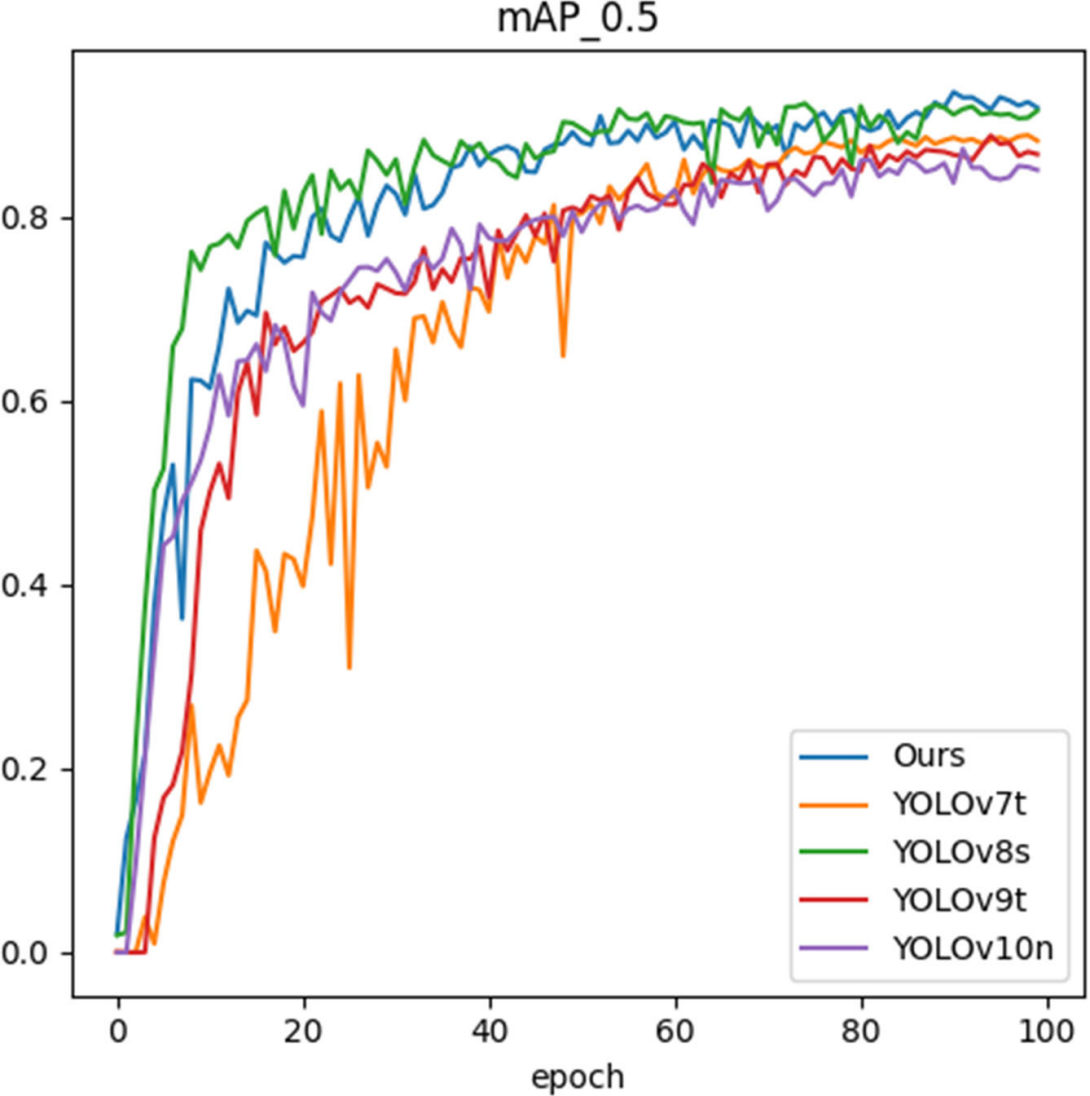
The mAP metrics of different models after different training epochs.

**Figure 12** shows the mAP metrics of different models after different training epochs. In the early stages of training, YOLOv8s has an initial advantage. However, as the number of epochs increases, our model gradually catches up and eventually surpasses it. As illustrated in **Table 2** and **Figure 12**, after training 100 epochs, our model slightly outperforms YOLOv8s in terms of mAP metric at the cost of lower GFLOPs and weight, indicating the efficiency and robustness of our model.

### 3.3 Comparison of heatmaps with YOLOv8s

**Figure 13:**
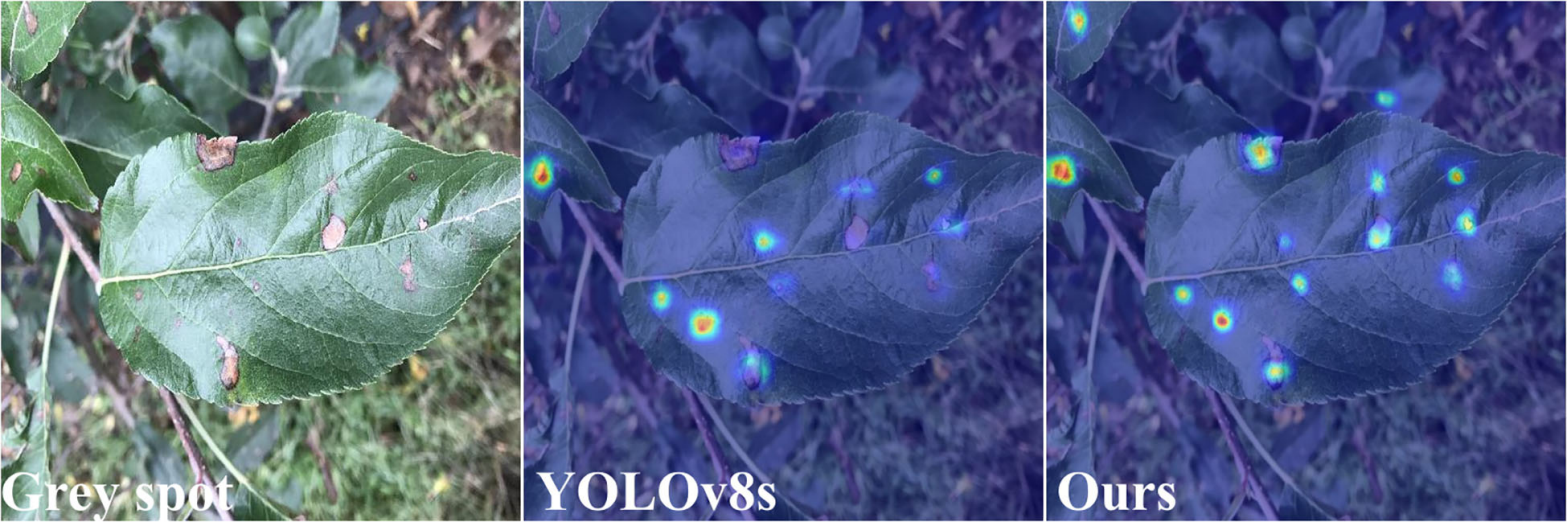
Heatmap-Grey spot.

**Figure 14:**
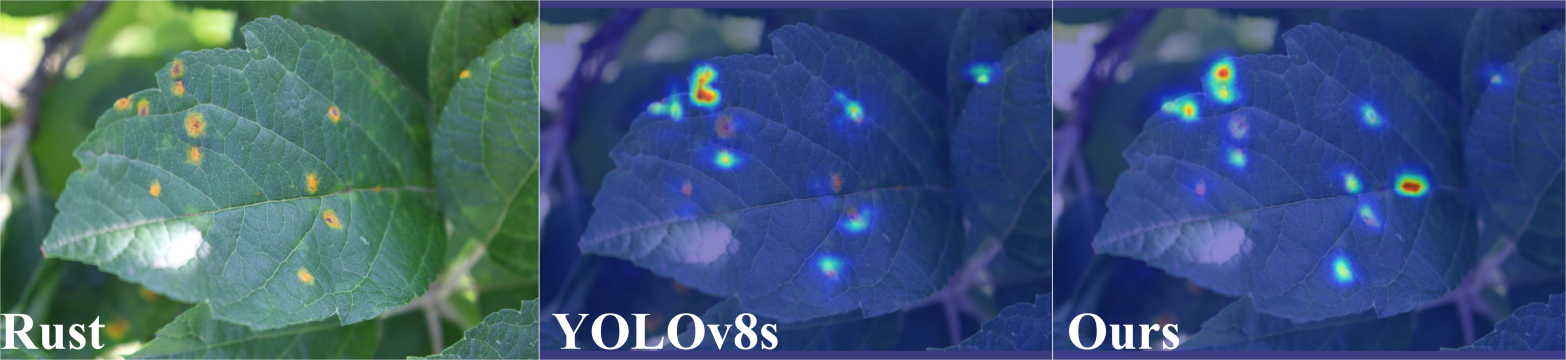
Heatmap-Rust.

**Figure 15:**
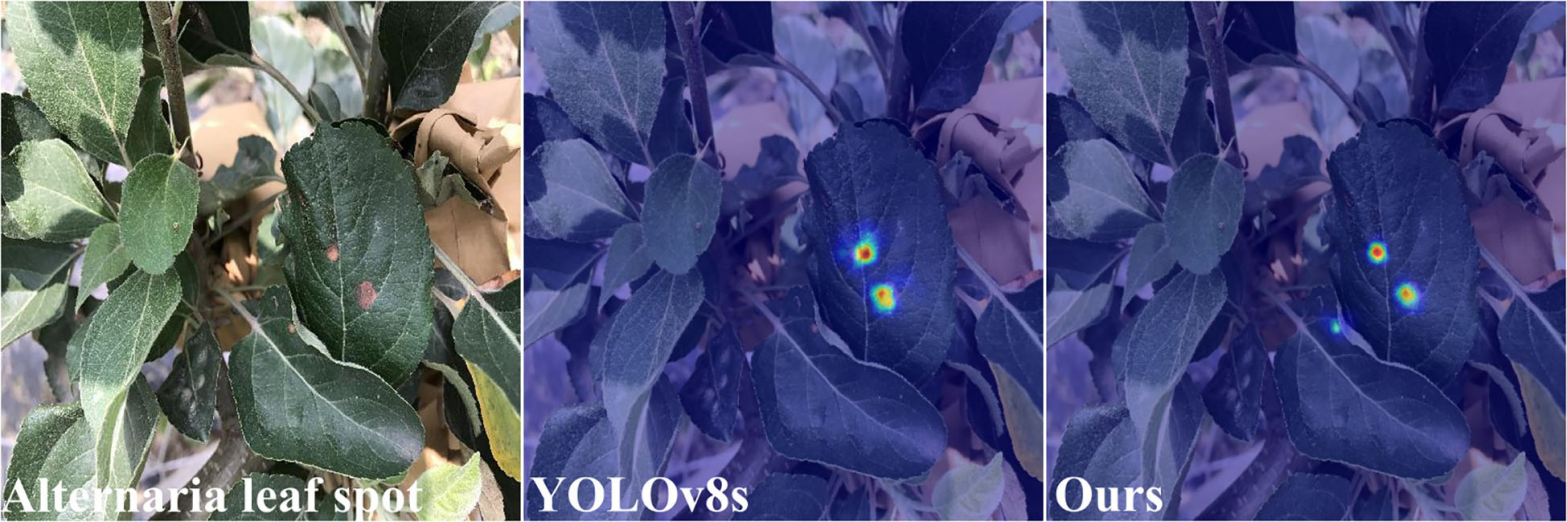
Heatmap-Alternaria leaf spot.

In **Figures 13**-**15**, we compare our model with the baseline model YOLOv8s through heatmap analysis. Our heatmaps were generated using the HiResCAM method, with the synthesis from multiple layers of the model. It is worth noting that the brown spot disease is not included because its characteristics are obvious, and all the comparative models can recognize it well. we observed that the YOLOv8s model was prone to false detection and misclassification when dealing with small target diseases. As shown in **Figures 13** and **14**, the heatmaps revealed that YOLOv8s sometimes failed to allocate sufficient attention for some small or obscured disease areas. The possible explanation was that the model gave more attention to larger targets during training but lacked adequate learning for smaller targets due to their less prominent features, low contrast, or occlusion.

Furthermore, YOLOv8s occasionally misclassified non-diseased regions as diseased ones, indicating its weak ability to distinguish features. In contrast, our proposed ALD-YOLO model can address these challenges better.

**Figure 16:**
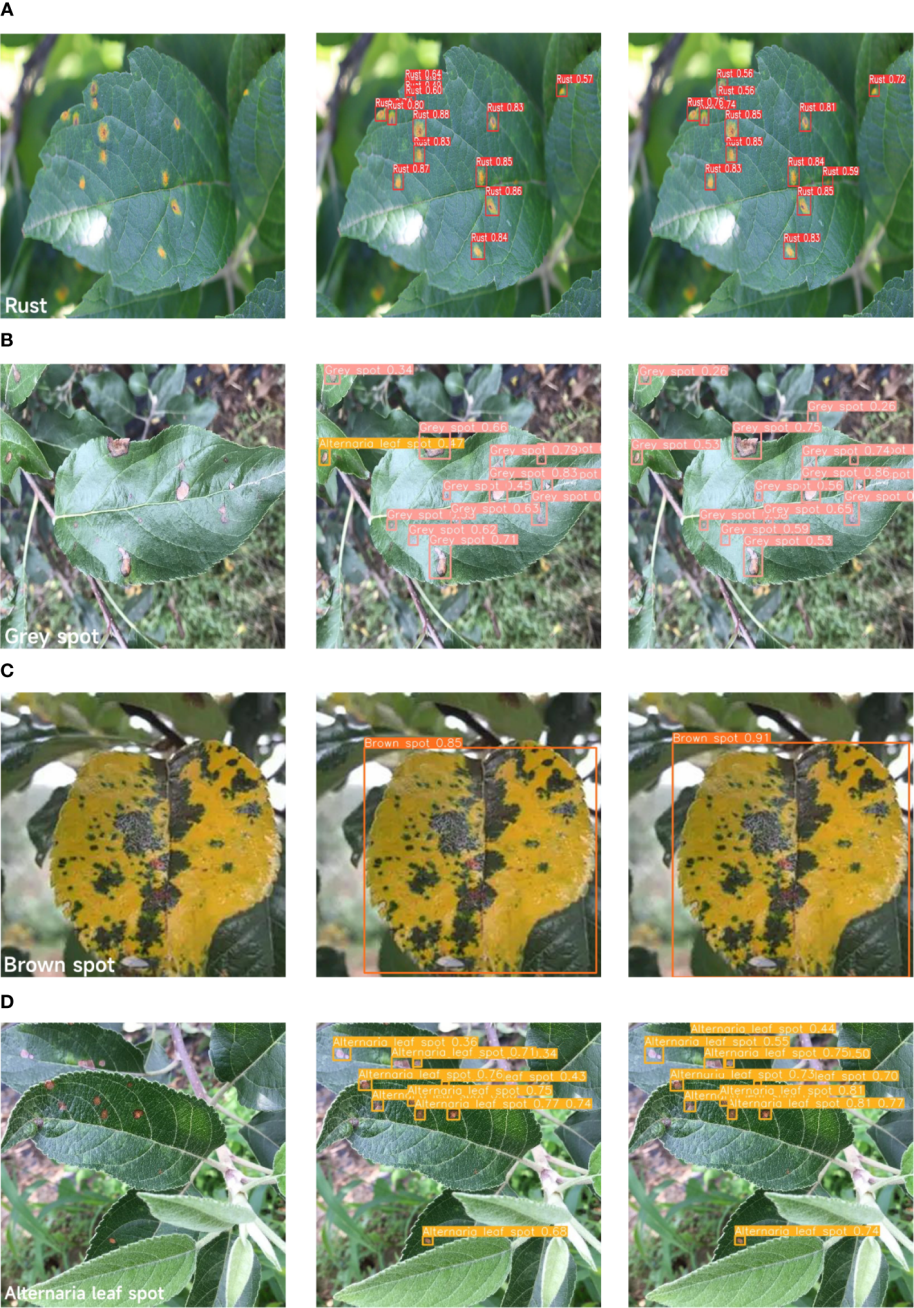
Model detection performance comparison. (**A**1–**D**1 respectively represent the original images of four different diseases. **A**2–**D**2 respectively represent the detection results inferred using the YOLOv8s model. **A**3–**D**3 respectively represent the detection results inferred using the ALD-YOLO model.).

In **Table 3**, YOLOv8s achieves the highest mAP for rust detection among the comparative models. Nevertheless, in certain scenarios, as shown in **Figure 16**, the YOLOv8 model has difficulties in detecting small targets and handling overlapping bounding boxes. In contrast, our proposed ALD-YOLO model can provide a more stable localization and identification of Rust disease on apple leaves while maintaining a similar detection accuracy. Moreover, in other categories of diseases, our model also ensures a certain advantage. Therefore, our model can effectively solve the problem of overlapping bounding boxes and detecting small targets.

### 3.4 Ablation experiment

Compared to the original YOLOv8s model, we achieved significant improvements in both computational efficiency and detection accuracy through a series of optimizations and modifications. To further investigate the roles of different modules, based on YOLOv8s, we designed four alternative models for comparison. **Table 4** lists different models, with checked marks indicating the inclusion of specific modules.

**Table 4:** Ablation experiment scheme.

Model	Faster_C2F	Faster_C2F_EMA	ADown
A	✓		
B		✓	
C			✓
D	✓	✓	
Ours	✓	✓	✓

Checked marks indicating the inclusion of specific modules.

**Table 5** presents the results of the ablation studies. Model A replaced all C2F modules in YOLOv8s with Faster_C2F. Compared with YOLOv8s, Model A showed a slight decrease in mAP@0.5, from 92.5% to 90.3%, but it reduced the computational load significantly, with GFLOPS dropping from 28 to 6.3. Thus, Model A can substantially enhance computational efficiency at the cost of slightly reducing the detection accuracy.

**Table 5:** Results of the ablation study.

Model	mAP@0.5	mAP@0.5-95	GFLOPS	Param	Weight
YOLOV8s	92.5%	63.1%	28.4	11.13M	21.481
A	90.6%	60.2%	6.3	2.30M	4.730
B	90.1%	59.5%	6.5	2.31M	4.781
C	91.9%	61.1%	7.4	2.59M	5.315
D	91.3%	60.6%	6.4	2.31M	4.759
Ours	93.1%	62.5%	5.7	1.89M	3.953

Model B further explored the effect of replacing all C2F modules with Faster_C2F_EMA. However, compared to Model A, Model B did not demonstrate any performance improvement, and there was no significant advantage in terms of parameter count or computational load (GFLOPS).

Model C achieved a mAP@0.5 of 91.9% by substituting the original Conv module in YOLOv8s with ADown.

Based on Model A, Model D replaced the C2F modules connected to the Head with Faster_C2F_EMA, Compared to Model A, with two different C2F modules, Model D improved mAP@0.5 from 90.6% to 91.3%, indicating that the suitable use of attention mechanism can improve the accuracy. From the beginning of our study, we always considered how to balance precision and efficiency, which is crucial in scenarios where the computational resources are limited. Therefore, based on experimental results, we only applied EMA to the selected C2F modules.

Finally, our proposed ALD-YOLO surpassed the original YOLOv8s with a mAP@0.5 of 93.1% by incorporating the ADown module into Model D. Moreover, GFLOPS and parameter count decreased significantly, reaching 5.7 GFLOPS and 1.89 M, respectively, suggesting that the ADown module can not only improve the model’s accuracy but also enhance the computational efficiency.

In summary, by combining two optimized C2F modules with an efficient downsampling module, our proposed model significantly reduces GFLOPS while maintaining high detection accuracy. Compared to the original YOLOv8s model, our model achieved a 0.6% increase in mAP@0.5, reduced GFLOPs from 28.4 to 5.7, decreased the parameter count from 11.13 M to 1.89 M, and lowered the weight from 21.481 M to 3.953 M, thereby offering better cost-effectiveness in practical applications.

In our task, the network backbone (Backbone) plays a crucial role in extracting feature representations from input images. The quality of these features directly determines the effectiveness of subsequent detection tasks. **Table 6** illustrates the performance differences among the backbone networks of various models.

**Table 6:** Comparison of backbone with other models.

Backbone	YOLOv8s	A	B	C	D	Ours
Params	5079712	911456	915824	993104	911456	631904
GFLOPs	12.59	2.16	2.26	2.72	2.16	1.65
Time (ms)	209.5	109.87	130.84	117.22	110.78	116.26

In **Table 6**, it can be observed that our model significantly improves computational efficiency and reduces resource consumption. Our model has only 631,904 parameters, far fewer than YOLOv8s’s 5,079,712, which not only reduces model complexity but also enhances deployment flexibility. Moreover, our model has a GFLOPs of merely 1.65, demonstrating a lower computational demand compared to other models. Time refers to the overall execution time of forward pass for the entire backbone when processing input images with 640x640 size. As shown in **Table 6**, the execution time does not scale linearly with GFLOPs. The reason is that the execution time is related to practical hardware constraints, such as parallel execution efficiency, memory bandwidth, and cache locality. However, GFLOPs is derived from a series of formulaic computations.

### 3.5 Inference testing

Considering that practical deployment scenarios may involve devices with limited computational resources, we conducted inference testing on two computers with different configurations. First, we deployed the model on a low-performance server using Ubuntu 22.04 system, an Intel Xeon Platinum CPU with a basic frequency of 2.5 GHz. Since this device lacked a graphics card, we performed inference using the CPU. Before each experiment, we have reduced the impact of background application interference to a minimum. and warmed up the device with a few rounds of test data to ensure CPU stability.

In **Table 7**, the YOLOv8s model achieves an inference speed of 2.6 frames per second (FPS), whereas our optimized model reaches 5.7 FPS with a 119.23% increase in speed. This significant improvement in speed makes our model applicable in a wider range of scenarios, especially on resource-constrained devices.

**Table 7:** Inference testing results.

Model	Platform	Weight(M)	Latency (s)	FPS
YOLOv8s	Ubuntu	21.5	0.38344	2.6
Ours	Ubuntu	3.9	0.17261	5.7
YOLOv8s	Windows	21.5	0.15933	6.3
Ours	Windows	3.9	0.09008	11.1

We also conducted the same test on another computer running Windows 11 and an Intel Core i5-13490F CPU with 4.2 GHz. Notably, all other settings remain consistent. The experimental results show that our model exhibits superior performance over the baseline model YOLOv8s on two computers with different operating systems and hardware configurations.

## 4 Discussion

In the traditional recognition of apple leaf diseases, there are issues of low efficiency and over-reliance on subjective judgment. To address these problems, we have proposed ALD-YOLO based on the YOLOv8 model. Through a series of optimization measures, we have significantly enhanced the model’s operational efficiency while ensuring its accuracy.

To ensure precise identification of apple leaf diseases under the natural conditions, this paper introduces an efficient apple leaf disease detection model based on multi-scale feature fusion. As shown in **Table 3**, our proposed model not only surpasses all comparative models in the mAP@0.5 metric, achieving the best performance, but it also maintains minimal complexity with respect to the number of model parameters and the size of weights. In the ablation experiments, as listed in **Table 5**, we further substantiate the efficacy of two novel C2f module in our ALD-YOLO model. Moreover, we have found that integrating an efficient downsampling module into the backbone and head components enhances the model’s computational efficiency without losing overall performance Compared to previous research, such as the method proposed by (Sun et al., 2024) et al., our ALD-YOLO achieves higher mAP for certain categories of diseases, along with a smaller model size and fewer FLOPs. This makes the model more suitable for deployment on edge computing devices, meeting the demands for high efficiency and low power consumption in agricultural IoT and similar scenarios.

However, as given in **Table 2**, the precision metric of our ALD-YOLO is slightly lower than that of YOLOv8s. The potential reason might be the lightweight design of our backbone. Generally, model lightweighting requires parameter compression, presenting inherent challenges in improving precision metrics. Consequently, in future research, we will focus on advancing efficient and lightweight backbone network.

## 5 Conclusions

Our ALD-YOLO model is built upon the YOLOv8 architecture for efficient detection of apple leaf diseases. In the Faster_C2F module, we have used the PConv to reduce redundant computations in feature maps, thereby maximizing the model’s efficiency. And in the Faster_C2F_EMA module, we further introduce the EMA attention mechanism, which utilizes multi-scale parallel sub-networks to enhance the model’s detection accuracy for diseased areas with varying sizes. The ADown module retains multi-scale spatial information during downsampling and reduces computational complexity.

By incorporating a series of optimization strategies aimed at achieving efficient and lightweight object detection, our model is designed to deliver superior performance while minimizing computational resources. Experimental results demonstrate that ALD-YOLO outperforms comparable models in terms of multiple metrics. Specifically, ALD-YOLO achieved a mAP of 93.1%, surpassing the original YOLOv8n’s 91.7% and slightly exceeding YOLOv8s’s 92.5%. Additionally, compared to YOLOv8s, our model’s GFLOPs significantly reduced from 28.4 to 5.7. In certain scenarios, ALD-YOLO also shows superior performance in small object detection. In the CPU inference tests, using two devices with different performance, our model achieved 5.7 FPS and 11.1 FPS, which is 76.19% and 119.23% faster than the original YOLOv8s model.

Furthermore, we identify several challenges when processing pathological datasets. In the actual process of detecting diseases in apple leaves, we have observed that certain leaf diseases may exhibit similar symptoms at different stages of development, such as Grey spot and Alternaria leaf spot, which increases the difficulty for the model to accurately distinguish between these diseases. Moreover, when multiple diseases occur simultaneously on the same leaf, this complexity can also adversely affect the accuracy of the model. To address these challenges, we will continue to optimize the network structure to enhance the model’s ability to handle these similar symptoms. Furthermore, we will expand our training dataset with more categories of disease images to improve the generalization ability of our model. In addition, we will continue to explore our lightweight model to foster its deployment on edge devices.

### Permission to reuse and copyright

Permission must be obtained for use of copyrighted material from other sources (including the web). Please note that it is compulsory to follow figure instructions.

### Resource identification initiative

To take part in the Resource Identification Initiative, please use the corresponding catalog number and RRID in your current manuscript. For more information about the project and for steps on how to search for an RRID, please click here.

## Data Availability

Publicly available datasets were analyzed in this study. This data can be found here: https://github.com/JasonYangCode/AppleLeaf9.
